# Toward a global harmonization of service infrastructure in academic clinical trial units: an international survey

**DOI:** 10.3389/fmed.2023.1252352

**Published:** 2023-10-12

**Authors:** Jean-Marc Hoffmann, Anette Blümle, Regina Grossmann, Henry Yau, Britta Lang, Cedric Bradbury

**Affiliations:** ^1^Clinical Trials Center, University of Zurich and University Hospital Zurich, Zurich, Switzerland; ^2^Clinical Trials Unit, Faculty of Medicine and Medical Center, University of Freiburg, Freiburg, Germany; ^3^Clinical Trials Centre, The University of Hong Kong, Pokfulam, Hong Kong SAR, China

**Keywords:** international harmonization, GCP, quality management, clinical research, academic research, organizational efficiency, university hospitals

## Abstract

**Background:**

Clinicians around the world perform clinical research in addition to their high workload. To meet the demands of high quality Investigator Initiated Trials (IITs), Clinical Trial Units (CTUs) (as part of Academic Research Institutions) are implemented worldwide. CTUs increasingly hold a key position in facilitating the international mutual acceptance of clinical research data by promoting clinical research practices and infrastructure according to international standards.

**Aim:**

In this project, we aimed to identify services that established and internationally operating CTUs – members of the International Clinical Trial Center Network (ICN) – consider most important to ensure the smooth processing of a clinical trial while meeting international standards. We thereby aim to drive international harmonization by providing emerging and growing CTUs with a resource for informed service range set-up.

**Methods:**

Following the AMEE Guide, we developed a questionnaire, addressing the perceived importance of different CTU services. Survey participants were senior representatives of CTUs and part of the ICN with long-term experience in their field and institution.

**Results:**

Services concerning quality and coordination of a research project were considered to be most essential, i.e., *Quality management*, *Monitoring* and *Project management*, followed by *Regulatory & Legal affairs*, *Education & Training,* and *Data management*. Operative services for conducting a research project, i.e., *Study Nurse with patient contact* and *Study Nurse without patient contact*, were considered to be least important.

**Conclusion:**

To balance the range of services offered while meeting high international standards of clinical research, emerging CTUs should focus on offering (quality) management services and expertise in regulatory and legal affairs. Additionally, education and training services are required to ensure clinicians are well trained on GCP and legislation. CTUs should evaluate whether the expertise and resources are available to offer operative services.

## Introduction

Clinical Trial Units (CTUs), as part of Academic Research Institutions (ARIs), are implemented worldwide and are considered to be a core element in enabling and coordinating excellence in academic clinical research (1, 2). CTUs aim to support clinicians at ARIs in conducting their own clinical research projects alongside their medical care responsibilities in daily practice. CTUs offer centralized administrative and organizational support and host conduct-related service infrastructure such as data management, vigilance, monitoring, biostatistics, and Information Technology (IT). The extent of services offered is a question of prioritization and depends on the locational requirements of the ARI and the resources available (3). Ever-evolving regulatory requirements and demands on valid clinical studies are likely to further consolidate CTUs within the clinical research environment in both number and extent.

CTUs have not only been shown to play a vital function in navigating clinicians through their respective national regulations and requirements, but they are additionally becoming addressees and drivers for the international harmonization of clinical practices as the international research community is facing challenges that call for transnational cooperation (1). On the one hand, the development of personalized healthcare demands that researchers tailor therapies to ever-smaller patient populations and rare diseases (4–6); on the other hand, health systems and their financing face the challenge of identifying effective and safe therapies for widespread diseases (7). New drivers in technical innovation such as artificial intelligence (AI) and Big Data add to the demand for cohesion (5, 8–10). However, expert knowledge and medical data distributed globally can only be coordinated and utilized in a targeted manner if we agree on common quality standards and research practices as well as standardized organizational practices following practical guidelines that transcend the conditions of heterogeneous legislations (11). The International Council for Harmonisation of Technical Requirements for Pharmaceuticals for Human Use (ICH) has been working since the mid-1990s toward achieving such greater international harmonization of clinical research by, for example, providing unified standards of Good Clinical Practice (ICH GCP) (12, 13). The aim is to ensure the safety of study participants as well as the quality and validity of the study results, thereby facilitating the mutual acceptance of clinical data by the regulatory authorities of different jurisdictions. The ICH guidelines today inform diverse national laws for drugs and medical devices, but they remain strictly formal. CTUs have a major responsibility in their local interpretation and practical application by developing and implementing an organizational service infrastructure that facilitates seamless and internationally standardized processes.

However, the identity of organizational services that emerging or growing CTUs should offer or prioritize to cater to the demands of high quality, internationally harmonized clinical research remains elusive. In the academic literature, recommendations for CTU service infrastructure remain scarce and at best singular rather than comprehensive (3, 14–16). Additionally, these publications are limited to the clinician’s (17, 18) as well as external stakeholder’s (1) perspective, or are outdated (19). The perspective of established, internationally operating CTUs has, to our knowledge, not yet been taken into account. The Swiss Clinical Trial Organisation (SCTO), a national CTU network in Switzerland, has published its Guidelines for Good Operational Practice, which are likely the most comprehensive resource available for informing (Swiss) CTUs about service and infrastructure requirements (20). In other countries, national authorities or stakeholder groups have published similar documents (21–25). These initiatives and documents – while adhering to international standards such as ICH-GCP – are however of limited range as they focus on translating into the respective national legal environment. The only dedicated International Clinical Trial Center Network (ICN) does not yet provide any pertinent resources to inform on CTU service infrastructure facilitating high quality, internationally harmonized clinical research (26).

With this paper, we aim to both identify the minimum set of CTU services that high profile internationally operating CTUs offer as well as to rank these services by their importance as perceived by these CTUs. The target group of our survey consists of representatives of CTUs, which are members of the ICN. The ICN is a non-commercial global network of high profile CTUs, whose goals are to enhance the global availability of high-quality clinical research centers and study sites, to enable advanced study centers to join forces, and to assist young study centers in building their clinical infrastructure and expertise. The Clinical Trials Centers in Zürich, Freiburg, and Hong Kong, which were involved in this project, are members of ICN and, with this paper, work toward promoting these goals. We herewith contribute to the global harmonization of organizational clinical research structures beyond the scope of ICH and provide both growing and emerging CTUs with a resource for scientifically informed service infrastructure implementation.

## Methods

### Survey conception

**Figure 1:**
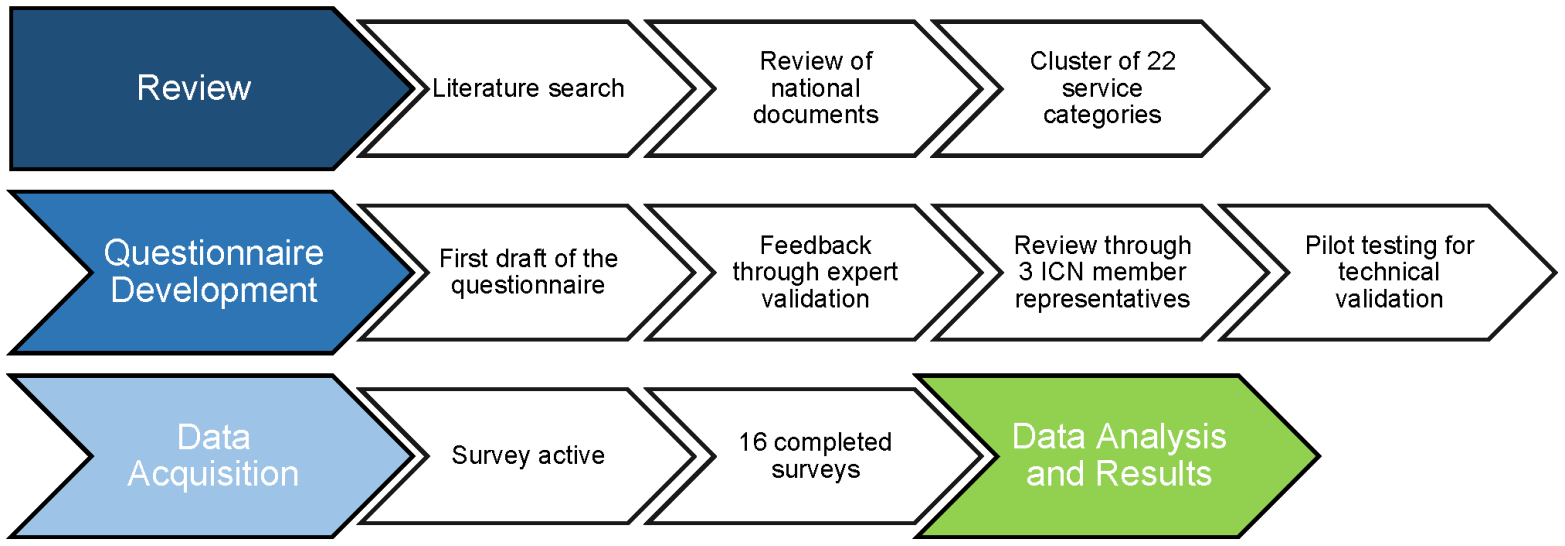
Workflow for survey conception to investigate the importance of various clinical trial units’ services.

To identify services that are relevant for the work of CTUs, we developed a questionnaire in English, according to the systematic seven-step design process recommended by the AMEE Guide (27). The setup-workflow of the survey is shown in Figure 1. Our approach takes into consideration both the previous knowledge revealed by a literature search and the expertise of international CTU members, i.e., of ICN. The questionnaire is provided as a supplement to this article (Supplementary File).

### Literature search

First, we conducted a literature search to identify prior research andexisting surveys assessing the importance of CTU services. We searched PubMed®^1^ using the following terms: Search 1: “CTU” or “Clinical Trial* Unit*”; Search 2: “Clinical Trial” and “Quality”; Search 3: “Clinical trial*” and “standard*”; “CTU” or “Clinical Trial* Unit*” and “service*.” We used the filter “Article Language” and selected English and limited the range of the publication date (custom range) to articles with the start date 01.01.2005; we manually excluded medical papers. A title and abstract screening of the remaining articles did not reveal any work addressing CTU services.

Second, we asked the representatives of all 24 CTU members of the ICN network by e-mail for national documents available in their country that make recommendations on CTU setup in regarding services offered. We sent two reminders and retrieved six national documents from five countries: Switzerland (20), Turkey (21), China (22, 23), the United Kingdom (24) and Australia (25). We started with the most extensive document from Switzerland and added the services recommended in the other documents incrementally. We then clustered the identified services into superordinate categories, which resulted in 22 service-categories.

### Questionnaire items

The 22 identified CTU service-categories were included in the questionnaire. For each service-category, participants were asked to indicate whether the service-category was “completely covered” by their CTU itself or “partly covered” (partly outsourced to either another clinic department or external third party) or “not covered” (completely outsourced to another clinic department or external third party).

To determine the perceived importance of each of the 22 CTU service-categories, we followed the importance scale ‘Essential’, ‘Quite important’, ‘Moderately important’, ‘Slightly important’, and ‘Not important’ as proposed by Artino et al. (27).

The response options for the importance scale depended on the answer given for the coverage as follows: if “complete coverage” was indicated, participants were asked: “How important do you consider these services for the effective work of your CTU?” If “partly covered” was checked, participants were asked: “Compared to other possible services, how important do you consider strengthening them is to increase the effectiveness of work of your CTU in the future?”; in a case of “no coverage,” they were asked: “Compared to other possible services, how important do you consider implementing them is to increase the effectiveness of work of your CTU in the future?.” This approach allows an assessment of the perceived importance of a service, regardless of whether the CTU offers the service or not. We collected the CTU’s basic characteristics including founding date, scope of services offered, number of employees (including full-time equivalents) and ratio of Investigator Initiated Trials (IITs) to Industry Sponsored Trials (ISTs) to determine possible correlations between the participating CTUs and the survey results.

We designed the questionnaire as an online survey in REDCap (28).

### Questionnaire validation

Seven experts in medical research and survey methodology validated the questionnaire. For pretesting, we sent the questionnaire to three ICN member representatives who did not participate in the survey and revised the questionnaire according to their feedback. Three persons who were not involved in the survey validated the functionality of the REDCap system.

### Survey population and conduct

We contacted all 24 CTU member representatives of the ICN via e-mail and asked them to participate in our survey. We asked for the contact details (name and e-mail address) of an appropriate person at their institution who would be qualified to answer the questionnaire as a representative of the CTU, but who had not been involved in the development of the survey. This person had to belong to the senior management staff, ensuring that the survey population consisted of senior representatives of CTUs with long-term experience in their field and in their institution. Participants were requested to refer in their responses to their own CTU, rather than to the organization to which the CTU is affiliated (e.g., University Medical Center).

In the original questionnaire, we did not collect personal information. To verify the expertise of our respondents and to be able to assess the reliability of the answers, we sent out a second questionnaire covering personal experience (asking about their position within CTU, years of involvement in clinical research, number of supervised studies). Since the answers also depend on the complexity of the trials conducted at a CTU, we also collected information on trial characteristics (percentage of drug vs. medical device vs. other studies, percentage of monocentric vs. multicentric studies, percentage of national vs. international studies). This second questionnaire is also provided as supplementary material (Supplementary File).

### Data management and analysis

We performed a general analysis of all services and analyzed in more detail the most ‘important’ as well as the ‘most unimportant’ services. We attributed a numerical value to each importance category (4 points for ‘Essential’, 3 points for ‘Quite important’, 2 points for ‘Moderately important’, 1 point for ‘Slightly important’, and 0 for ‘Not important’) and added up the values to receive a final score for each service. This points distribution was necessary to make a comparison and rating amongst the different importance categories. This resulted in a list of 22 services ranked by perceived importance for the effective functioning of a CTU as seen by an international cohort of high-profile Clinical Trial Units. The quantitative analysis was carried out through standard descriptive methods using Microsoft Excel (Microsoft Corporation, Redmond, Washington, United States: Version 16).

The reporting of this survey conforms with the CROSS guideline (29).

## Results

We analyzed 15 responses out of the 16 received. We excluded one CTU due to self-contradictory statements (detailed explanation in the Supplementary File). Twelve CTUs (80%) offered both study coordination and study execution including patient contact, while three CTUs (20%) offered study coordination only (without patient contact). Characteristics of the participating CTUs are shown in Table 1.

**Table 1:** Characteristics of the participating CTUs: **(A)** founding date, organization, country; **(B)** number of employees, percentage of performed IITs in 2020 and types of performed clinical trials; **(C)** personal experience of the CTU representative (XX = missing information).

**(A)**		
Founding date	Organization	Country
2013	Academic Clinical Research Office, Khon Kaen University	Thailand
1993	Baim Institute for Clinical Research, Boston	USA
1997	Clinical Trials Unit, Medical Center – University of Freiburg	Germany
2006	University of South Australia, Adelaide	Australia
2006	Clinical Trials Center, University Hospital Zurich	Switzerland
2012	Clinical Trial Coordination Centre, Medical University of Graz	Austria
2008	Shanghai Clinical Research Center	China
1998	Clinical Trials Centre, The University of Hong Kong	Hong Kong
1952	Oncology Clinical Trials Unit, University College Hospital Ibadan	Nigeria
2001	Münchner Studienzentrum, Technical University of Munich	Germany
2006	Clinical Trial Center, Department of Research, Hualien Tzu Chi General Hospital	Taiwan
2006	Centre for Clinical Trials, Essen	Germany
2011	Cambridge Clinical Trials Unit, Cambridge University	United Kingdom
1999	Institute for Advancement of Clinical and Translational Science, Kyoto University	Japan
2002	Infectious Diseases Institute, Kampala	Uganda

**(B)**				
Number of employees	Percentage of studies			
	IITs out of all studies (IITs + ISTs) in 2020	Pharmaceutical / medical device/other research	Monocentric / multicentric	National / international
8	95	40/50 / 10	90 / 10	95 / 5
42	95	30 / 10 / 60	60 / 40	70 / 30
260	90	15 / 0 / 85	62 / 38	69 / 31
30	15	60 / 0 / 40	20 / 80	90 / 10
10	5	85 / 5 / 10	20 / 80	30 / 70
32	90	5 / 5 / 90	65 / 35	95 / 5
24	94	55 / 14 / 31	14 / 86	86 / 14
85	30	XX / XX / XX	XX / XX	XX / XX
130	100	84 / 6 / 10	11 / 89	51 / 49
112	41	XX / XX / XX	XX / XX	15 / 85
9	35	80–90 / 2–5 / 5–8	70 / 30	80–90 / 10–20
87	100	45 / 6 / 49	16 / 84	52 / 48
60	70	80 / 10 / 10	40 / 60	90 / 10
26	5	80 / 1 / 19	<1 / 99	5 / 95
100	100	66 / 5 / 29	10 / 90	93 / 7

**(C)**		
Position	Years of involvement in clinical research	Involvement in study design, planning and conduct? If yes, how many supervised/supported?
Managing director	20	Yes, 50
Deputy director of clinical operation department	10	Yes, as leader of study planning and conduct process, over 20
Consultant, clinical pharmacology and clinical trials expert	23	Yes, over 40
Managing director	25	Yes, hundreds
Project manager	10	Yes, 20
Managing director	22	Yes, at the beginning as study coordinator, then as project manager, now as head, XX
Quality management representative	23	Not on an operational basis but on a Quality assurance function and more in the sense of overall planning and assuring correct (according to regulations and ICH GCP) conduct, around 120
Clinical data manager	5	No, Review of study documents for eCRF setup
Operations director	15	Yes, in an advisory and oversight capacity, multiple
Unit chief for international collaboration	22	Yes, 30
Director of trial design and development	15	Yes, XX
Managing director / deputy managing director	22 / 16	Yes, over 100
Managing director	25	Yes, over 50
Dean of research	20	Yes, about 25
Trial manager and clinical trials monitor, medical doctor	23	Yes, over 20

Different order for the CTUs in table A, B, and C in order to adhere to our data privacy policy.

The CTUs were founded between 1952 and 2013 with a median founding year of 2006. The median number of employees was 42 and the average number 68, with a maximum of 260 and a minimum of 8. The estimated percentage of the coordinated IITs out of all studies (i.e., IITs plus ISTs) from January to December 2020 reached a median of 90% and an average of 64%. Two CTUs (13%) conducted a minimum of only 5% IITs, whereas a maximum of 100% was reached by three CTUs (20%). Seven CTUs (47%) conducted 80% or more multicentric studies and all CTUs conducted studies on an international level.

### Analysis of the services provided by the CTUs

**Figure 2:**
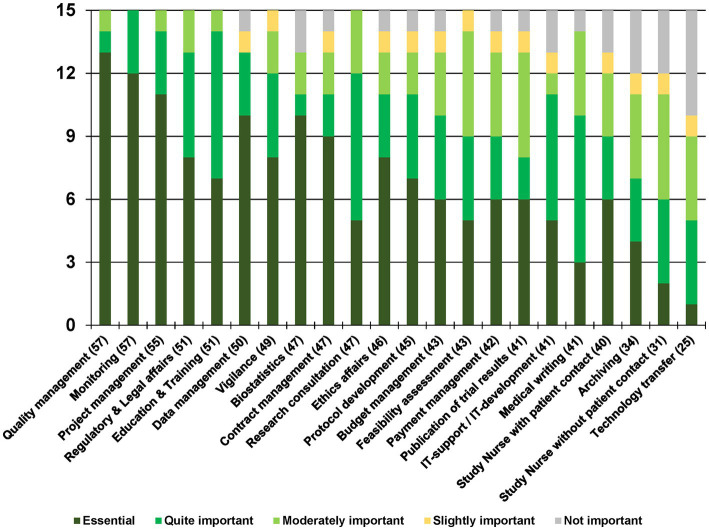
Overview of the rating of the 22 service categories. The number in brackets next to each service category represents the total score.

The rankings of the 15 CTUs for each of the 22 service categories concerning their importance is shown in Figure 2. For example, *Monitoring* was considered by all 15 CTUs (100%) as important (12x essential, 3x quite important), *Technology transfer* only by five (33%) (1x essential, 4x quite important).

*Quality management* was ranked as the most important service with 57 points (60 being the highest possible score) and 13 ‘Essential’ votes. *Monitoring* also received 57 points with 12 ‘Essential’ votes, followed by *Project management* with 55 points and 11 ‘Essential’ votes. *Regulatory & Legal affairs* and *Education & Training* with 51 points, along with *Data management* with 50 points, made it to the top of the ranking list.

The two services related to performing study visits – *Study Nurse with patient contact* and *Study Nurse without patient contact* – were ranked lower, with 40 and 31 points, respectively; while *Technology transfer*, with 25 points, was considered as essential only by one CTU (7%).

We attributed the CTUs into two groups depending on the percentage of performed international trials: the first group contained the CTUs performing mostly national trials (i.e., international studies only up to 20%), *n* = 7; the second group contained the CTUs performing mostly international trials (i.e., international studies >20%), *n* = 7. One CTU did not provide us with details on their percentage of performed national/international trials. We compared the average score (rating between 4 and 0 points) attributed to each service by the two groups using the nonparametric Mann–Whitney U test due to the small sample size and non-normal distribution. The ratings of all the services were not significantly different between the two groups.

Table 2 shows the coverage of each service by the respondents as well as their total score. *Quality management* for example was provided by 93%, or 14 out of 15 CTUs. Instead, *Technology transfer* was the most ‘Not covered’ service with only 20%, or three out of 15 CTUs, offering it. Two CTUs (13%) not offering *Technology transfer* considered this service ‘Quite important’, however. Similarly, two CTUs not providing *Medical writing* defined this service nevertheless as ‘Quite important’. Five CTUs (33%) considered *Technology transfer* a service not worth offering; of those, four (27%) did not cover this service at their CTU at all, and only one (7%) only partly did. Similar relations were shown for the other services, *Archiving*, *Study Nurse without patient contact*, *Biostatistics*, *IT-support / IT-development* and *Study nurse with patient contact*. None of the services was covered in full by a CTU.

**Table 2:** Overview of the coverage of the 22 service categories.

Services	Coverage						Score
	Covered		Partly covered		Not covered		
	%	Number	%	Number	%	Number	
Quality management	93	14	7	1	0	0	57
Monitoring	87	13	7	1	7	1	57
Data management	87	13	0	0	13	2	50
Project management	80	12	13	2	7	1	55
Regulatory and legal affairs	80	12	7	1	13	2	51
Education and training	80	12	20	3	0	0	51
Biostatistics	80	12	0	0	20	3	47
Research consultation	80	12	20	3	0	0	47
Vigilance	73	11	13	2	13	2	49
Contract management	73	11	13	2	13	2	47
Protocol development	73	11	20	3	7	1	45
Publication of trial results	73	11	13	2	13	2	41
Ethics affairs	67	10	13	2	20	3	46
Feasibility assessment	67	10	33	5	0	0	43
Payment management	67	10	13	2	20	3	42
Archiving	67	10	20	3	13	2	34
Budget management	60	9	33	5	7	1	43
IT-support / IT-development	60	9	20	3	20	3	41
Study nurse with patient contact	60	9	20	3	20	3	40
Medical writing	53	8	20	3	27	4	41
Study nurse without patient contact	33	5	20	3	47	7	31
Technology transfer	20	3	27	4	53	8	25

### Detailed analysis of services needed for the conduct of study visits

**Figure 3:**
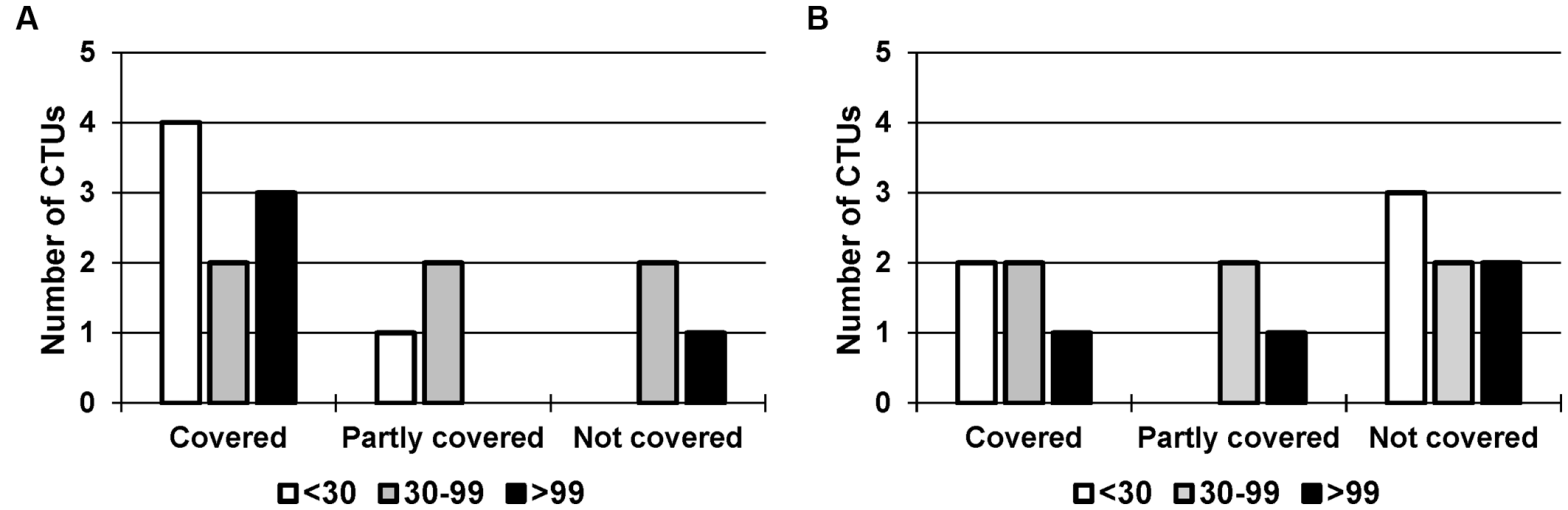
Coverage of the service: **(A)** Study Nurse with patient contact vs. **(B)** Study Nurse without patient contact, depending on the number of employees. CTUs with <30 employees in white (*n* = 5), 30–99 employees in grey (*n* = 6) and > 99 employees in black (*n* = 4).

To gain more insight into services linked to performing study visits, we took a closer look at those CTU services regarding their association of coverage with the judgment of importance (Table 3) and with the number of employees (Figure 3).

**Table 3:** Coverage of the service: **(A)** Study nurse with patient contact vs. **(B)** Study nurse without patient contact.

**(A)**						
Study Nurse with patient contact	Essential	Quite important	Moderately important	Slightly important	Not important	Total
Covered	6	3	0	0	0	9
Partly covered	0	0	2	1	0	3
Not covered	0	0	1	0	2	3
Total	6	3	3	1	2	15

**(B)**						
Study Nurse without patient contact	Essential	Quite important	Moderately important	Slightly important	Not important	Total
Covered	1	2	2	0	0	5
Partly covered	0	1	2	0	0	3
Not covered	1	1	1	1	3	7
Total	2	4	5	1	3	15

The service *Study Nurse with patient contact* was reported as being covered by nine participants (60%), as partially covered by three participants (20%), and by another three as not covered (20%). Out of the 15 respondents taking part in the survey, six (40%) regarded this service as ‘Essential’, three (20%) as ‘Quite important’, and they all covered this service. At CTUs without or partly covering this service, it was considered less relevant (moderately, slightly, not important). In contrast, the service *Study Nurse without patient contact* was less often reported by the participants as covered (‘Covered’ by five (33%), ‘Partly covered’ by three (20%)). Two of 15 (13%) respondents ranked this service as ‘Essential’, one did not currently provide the service, another nine (60%) considered it ‘Quite important’.

We analyzed the coverage of the services *Study Nurse with patient contact* and *Study Nurse without patient contact* in relation to the number of employees for the following three categories: <30 employees (*n* = 5), 30–99 employees (*n* = 6), and > 99 employees (*n* = 4). Among the smaller CTUs (<30 employees), 80% covered the service *Study Nurse with patient contact* and 40% the service *Study Nurse without patient contact*. For CTUs with 30–99 employees, both services were covered by 33%; for CTUs with more than 99 employees, 75% offered the service *Study Nurse with patient contact* and 25% the service *Study Nurse without patient contact*.

We performed the Freeman–Halton extension to Fisher’s exact test. With a *value of p* of 0.60 and 0.83 for the services *Study Nurse with patient contact* and *Study Nurse without patient contact*, respectively, we could assume that the two variables ‘coverage’ and ‘number of employees’ were independent. The number of employees had no effect on whether the services were covered or not.

## Discussion

The main finding of our survey is that according to our respondents, services concerning quality and coordination of a research project are considered to be the most essential: *Quality management* (57 points), *Monitoring* (57 points) and *Project management* (55 points). The top three services were followed by *Regulatory & Legal affairs* (51 points), *Education & Training* (51 points) and *Data management* (50 points). To put a cut-off line after a certain number of services would be arbitrary. However, when considering that a service qualifies as ‘Quite important’ once it reaches a sum of 45 points (on average 3 points from each respondent), *Vigilance* (49 points), *Biostatistics* (47 points), *Contract management* (47 points), *Research consultation* (47 points), *Ethics affairs* (46 points) and *Protocol development* (45 points) should also be taken into account by new CTUs.

To our surprise, the operative services for conducting a research project, *Study Nurse with patient contact* was fourth-last (40 points) and *Study Nurse without patient contact* second-last (31 points) on our list. The question arises whether emerging CTUs should focus on performing study visits or not. Fleischmann et al. analyzed why doing study visits is interesting for physicians: (1) physician-investigators can apply their medical knowledge in a research setting and create new findings, and (2) the conduct of study visits on behalf of external sponsors is financially rewarding (17, 18). From a CTU perspective, the answer is not so clear. The majority of our respondents provide services for the conduct of study visits, but they do not consider this service to be very important during their daily work. Potential reasons against conducting study visits could be as follows: first, a high time expenditure is associated with each visit; second, study nurses and doctors need to familiarize themselves with the subject matter of the study (on a scientific/medical level); third, the CTUs need qualified personnel with a corresponding medical background; and fourth, the medical knowledge gained during the study visits cannot be transferred as easily to studies of a different medical specialty as for example general management skills. Moreover, due to outsourcing of the study visits to specialized CTUs, the study visit costs might increase for the sponsor (30–32). For IITs, the clinicians themselves could perform the study visits as they have the medical knowledge and practice in assessing the benefit of a treatment, and thus they are most efficient. Consequently, the sponsors lower the costs for the required CTU services. However, clinicians need to have good knowledge of current GCP practice. Training and refreshers through CTUs are important and should not be neglected.

The service *Archiving* was reported by 10 participants as ‘covered’ (67%) and by three participants as ‘partly covered’ (20%), while at the same time being ranked low on the importance scale. Archiving, even though sometimes tedious, is mandatory according to GCP. Archives need to ensure the long-term storage of data for up to 15 years. Storage rooms can either be made available through CTUs or through the clinics themselves. The services *Medical writing* and *Publication of trial results* can be performed by CTUs. However, close contact with clinicians is necessary to rely on their expertise about a specific medical condition. Consequently, sponsors or principal investigators might prefer to write the manuscripts themselves because it is intellectually rewarding and to save costs/be more efficient. The service *Technology transfer* has to be considered separately from the other 21 services listed. In fact, lawyers and specialized contract managers need to be involved to spin off a company based on a research project. Most academic institutions centrally manage this under a separate unit (e.g., Technology Transfer Office or equivalent). While it may not be important to CTUs, it does not mean that it is unimportant to the wider institutions to which they are affiliated. For institutions that only focus on ISTs, intellectual property (IP) protection and transfer is not an area that is worthy of attention because IP ownership belongs to sponsors.

CTU services required by investigators for IITs and ISTs are very different. For ISTs, investigators mainly focus on protocol compliance concerning study procedures and data collection, and do not need to concern themselves too much about study design and protocol development, regulatory and legal affairs, project management, monitoring, data management, biostatistics and even manuscript writing for publication. CTUs focusing on ISTs target facilitating and accelerating collaboration between local investigators and sponsors/CROs, and therefore put services such as feasibility assessment, ethics submission, budget and payment management, contract management and archiving as priorities.

In contrast, IITs are organized independently from commercial sponsors/CROs, therefore investigators ask their CTUs to provide services on a broader scope, such as regulatory and legal affairs, project management, monitoring, data management and biostatistics. Most investigators prefer to take charge of the intellectual elements of their IITs – including study design and protocol development, grant application and management as well as manuscript writing for publication.

Table 1 shows that most of the survey respondents focused on IITs. In fact, nine out of the 15 responding CTUs focused fully or mainly on IITs. This explains why services such as regulatory and legal affairs, project management, monitoring, data management and biostatistics rank higher in our survey. We therefore recommend emerging CTUs consider their institutions’ research focus (i.e., IITs and/or ISTs) to determine the priorities of their service.

Conducting a clinical trial involves not only scientific but ethical aspects. In fact, only through completion of the study and publication of the results can the scientific community and patients profit from the newly gained knowledge (33). Ensuring the completion and good conduct of clinical research projects is vital, as stated in the Declaration of Helsinki (34). Sponsors therefore need to conduct their studies according to highly regulated GCP principles. Especially for IITs where sponsors and principal investigators perform studies on top of their regular clinical duties, this management needs to be assured. As shown by Marchesi et al., the implementation of a CTU within the Italian Sarcoma Group in 2010 led to a fivefold increase in clinical studies within 5 years (15). Medical doctors are experts in treating patients, however, they may lack experience in the conduct/management of clinical research projects. Inefficiencies in study conduct such as failure to meet recruitment targets or poor data management as reported by Duley et al. need to be minimized (14). In fact, specialized CTUs try to increase conduct efficiency by offering GCP training and management support.

Clinical research projects have to undergo regular audits and inspections. Common deficiencies observed by the US Food and Drug Administration included inadequate record keeping, protocol deviations, or failure to follow the investigational plan (35, 36). Advantages linked to involving a CTU with an experienced team include improved quality assurance, a standardized project management system, faster trial development, and higher participant accrual (30, 37). Education and training carried out by the CTUs leads to better-educated sponsors and investigators and consequently improved quality conduct of clinical research projects (3, 38, 39).

For emerging CTUs the initial scoping – building on existing links with national and international trial units – is most important. This collaboration, as described by Brown et al. (16) is vital in order to develop successful processes. Our survey tries to help CTUs during this initial scoping phase by developing a guide to assess which services are most essential.

A limitation of our study is the focus on the ICN itself, resulting in the small sample size of 15 respondents. The extent of membership activity within the ICN is voluntary and at the member’s own discretion. Under these conditions, we consider a response rate of 67% (16 out of 24) and a usefulness rate of 15 out of 16 answers satisfactory. Since, as described, the answers were very comparable to one another and we observed only few significant outliers, we assume that the statements made are to a high extent representative of the ICN as a whole. While the ICN has strict membership criteria, it is neither the single point of contact to high profile and internationally operating CTUs, nor does it hold sovereignty of interpretation on the topic explored in this study. On the other hand, our restriction to the ICN made for a very clearly delineated study population and prevented a selection bias. Additionally, the responding CTUs are internationally distributed over five continents, thus allowing us to include different perspectives from around the world. Nevertheless, the present work is exploratory in nature and should trigger further in-depth and large-scale research on this topic beyond the opinion space of the ICN.

We intentionally refrained from giving a definition for each service, which led to a certain heterogeneity. However, at the same time the room for interpretation allows a better overview of the prioritization. As the results of the survey have subjective components – based on what has been reported by the participants – potential self-reported bias and response errors cannot be excluded. One participant was less-experienced in clinical research, compared to the other respondents, by working as a clinical data manager for only 5 years. However, as this participant was working in a CTU with long-lasting experience in academic clinical trials and having as data manager a good overview of the other CTU services, we considered the answers trustworthy. We used the Freeman–Halton extension to Fisher’s exact test, assuming a directional hypothesis and that the answers given by the respondents were independent from each other.

In summary, emerging CTUs should focus on management services, regulatory and legal affairs, as well as education and training. Qualified clinicians and study personnel are needed to plan, conduct, and evaluate the research projects in a qualitatively robust way. For this reason, education is particularly important. It is the responsibility of the CTUs to continuously update the content of the training with the latest local, national, and international innovations so that the researchers are up to date. This is especially important for new trends such as ‘digital research’, Big Data and AI approaches. For such projects, even if GCP is not always directly applied, an understanding of the quality and validity of the data is essential. Clinicians perform academic clinical trials in order to establish new and safe treatment options for rare and widespread diseases, independently of pharmaceutical companies, however, they need guidance through specialized CTU professionals in order to be GCP-compliant and conform to international standards.

Moreover, each CTU should evaluate whether they have the expertise and the resources to perform study visits themselves or not. Most important is a good collaboration between CTUs and sponsors, investigators, and clinicians. This cooperation leads to GCP-compliant conduct of studies, keeps the study costs reasonable and profits both researchers and the public.

We feel that there should be international efforts, similar to ICH, to practically harmonize trial processes and trial-related services of CTUs. The ICN plays an important role in driving such an initiative. With this paper, we provide a focused resource that can scientifically inform growing and emerging CTUs on their quest to implement a service infrastructure that meets modern standards for high quality, internationally harmonized clinical research.

## Data availability statement

The original contributions presented in the study are included in the article/Supplementary material, further inquiries can be directed to the corresponding author/s.

## Group members of ICN-Quality Standards working group

Bauer, A., Eskat, A., Hiemstra, T., Kaufmann, C., de Medina Redondo, M., Üresin, Y., Şen, S., Stockley, L., Wan, M., and Wang, T.

## Author contributions

CB and BL contributed to the development of methods and questionnaire. CB performed a literature search and conducted the survey. J-MH, CB, and AB performed the data analysis and visualization and wrote the first draft of the manuscript. RG, HY, BL, CB, AB, and J-MH wrote the sections of the manuscript. All authors contributed to manuscript revision, read, and approved the submitted version.

## Footnotes

^1^https://www.ncbi.nlm.nih.gov
