# Supplementary material for: Toward a global harmonization of service infrastructure in academic clinical trial units: an international survey
Source: Front Med (Lausanne). 2023 Oct 12;10:1252352. doi: 10.3389/fmed.2023.1252352 (PMC10602721; doi:10.3389/fmed.2023.1252352)
Supplement: Supplementary file 1 [file Data_Sheet_1.PDF]

ICN Survey: Services of Clinical Trials Units

Dear ICN-member,

Dear survey participant,

Using the experience of your established facility we would like to identify a minimum set of services that (emerging) Clinical Trials Units (CTUs) should cover to ensure comprehensive and smooth working processes.

The following survey aims to ascertain which fields of activity and services are covered by your facility and what importance you attribute to these areas for the work of your CTU/CTC.

For this purpose we would very much appreciate if you could invest 10 minutes of your time to answer the 4 sets of questions below.

Important practical information:

- The questionnaire should be completed by senior management staff of your facility.

- Your CTU representative should not have participated in the development of this survey.

- The questions aim at surveying the services of your facility, not the services of the parent institution (e.g. University Hospital).

- The survey can be interrupted and continued at any time.

- You can go back at any time in the questionnaire to review and change entries before submitting.

- Starting from page 3 of the survey you will be asked whether your CTC/CTU "completely covers", "not covers" or "partly covers" certain areas of work. "Partly covered" refers to regularly or occasionaly outsourcing otherwise covered tasks to external parties due to capacity constraints.

- If you feel the need for clarification or differentiation of your answers, feel free to use the provided text fields.

- The results of this survey may be published in a scientific journal.

- Before starting the survey, first read the data privacy policy below.

Thank you!

Contact: cedric.bradbury@uniklinik-freiburg.de

Data Privacy Policy and/or Declaration of data protection consent for a survey on the subject of "Services of Clinical Trials Units"

On the following pages, we would like to ask you a few questions about the services your Clinical Trials Unit (CTU) or Clinical Trials Center (CTC) provides. The aim of our survey is to be able to evaluate better the importance of individual services for the work of a CTU/CTC.

At no point in this questionnaire are you asked to provide personal information. For reasons of data protection, we would like to point out that you should not enter any direct personal data of other persons or your own person (such as your own name, your own function in the organization) in the free text fields of this questionnaire. Definitions:

- Survey data: Data, that we want to collect for the described purpose above. Survey data are in the survey database.

- Survey distribution data: Personal data, that are necessary to collect the survey data. In particular, your email address and personalized survey distribution link. Survey distribution data are stored in the survey distribution database. Survey distribution data are not intended for export.

We cannot ensure, that data in the survey database stored in anonymous form, because of the following reasons:

1. Since only a small amount of CTUs/CTCs are surveyed and the survey participants are known, we cannot ensure that the data in the survey database are in anonymous form due to unique responses to be made on page 2 and 3 of this questionnaire.

2. We created a personalized survey distribution link for you to access the questionnaire, to prevent multiple completion of the questionnaire through the same CTUs/CTCs. We use your email address to send you the personalized survey distribution link. Although your personalized survey distribution link and your email address are not stored in the survey database, your email address will be stored permanently in the survey distribution database. The email address will be stored at the survey distribution database, until the data in the two databases (survey database, survey distribution database) is deleted. The two databases will be deleted after the survey is finalized and after the survey data is exported.

3. Your personalized survey distribution link will be stored in the survey distribution database for as long as you have not completed the questionnaire and until the survey distribution data is deleted, as described under point 2.

Therefore, once the data in the survey database has been exported, the data will not contain personalized survey distribution links, email addresses or other identifying data. Nevertheless, we cannot ensure, that the exported data are in anonymous form, because after the export the reason of point 1 (see above) persist. Once all attendees completed the survey, the survey data will be exported and after that, all data (survey data and survey distribution data) will be deleted on the survey IT system. The exported survey data will be stored at the central file server of the Universitaetsklinikum Freiburg with restricted access. Only certain employees of the Zentrum Klinische Studien of the Universitaetsklinikum Freiburg have access to the survey data.

The answered and transferred questionnaires are stored on servers at the Universitaetsklinikum Freiburg. The Universitaetsklinikum Freiburg is allowed to transfer selected survey data to scientific cooperation partners like e.g. ICN members or professional journals, without restrictions for the scientific cooperation partners.

The scientific cooperation partners may be located in third countries outside the European Union. For the third countries it is not assured, that there is an adequacy decision by the European Commission, which confirms an appropriate data protection level comparable to that of the European Union. The third countries may provide no appropriate or suitable data protection level or data protection safeguards. For example, governments or private entities may have access to your data, although this is not allowed through data protection regulations of the European Union. Your rights of person affected may be limited and not as enforceable as within the European Union.

The legal basis for your consent is Art. 6 (1) a GDPR (General Data Protection Regulation). For the transfer to a third country without guarantees in conjunction with Art. 49 (1) a GDPR.

The contact information of the data protection officer of the Universitaetsklinikum Freiburg are:

Universitaetsklinikum Freiburg
Datenschutzbeauftragter
Breisacher Straße 153
79110 Freiburg
Germany

If you have further questions about data protection, please contact the data controller or the data protection officer.

Your exported survey data is stored as long as the survey data seems useful for the purposes they have been collected. There is no predetermed time for the deletion of the exported survey data.

Rights of data subject:

a) You have the right to withdraw your consent for the data processing at any time, without affecting the lawfulness of processing based on consent before its withdrawal (Art. 7 (3) GDPR). If you withdraw your consent, your data will be deleted without undue delay.

b) You have the right to request erasure of your personal data (Art. 17 GDPR).

c) You have the right to request from the controller access to your personal data (Art. 15 GDPR).

d) You have the right to request data portability (Art. 20 GDPR).

e) You have the right to request rectification of your personal data (Art. 16 GDPR).

f) You have the right to request restriction of processing of your personal data (Art. 18 GDPR).

Nevertheless, please be aware, that your rights may be restricted because although you data are not anonymous (see points 1, 2 and 3 above), we probably cannot sure identify your survey data in case:

- Some of the survey distribution data already deleted.

- We cannot link your survey distribution data set to a single survey data set (for example, if two or more attendees of the survey already completed the survey).

Also, as mentioned above at point 2, your email address cannot be deleted from the survey distribution data base, until the survey of the attendees is completed and until the survey distribution data is deleted on the survey IT system after the survey data export.

If you want to exercise your rights (mentioned at points a, b, c, d, e, and f above) as a data subject, please contact the data controller or the data protection officer.

You have the right to lodge a complaint with a supervisory data protection authority (Art. 77 GDPR).

Please check the checkbox below to agree with the Data Privacy Policy and to consent with the data collection described above and to continue with the questionnaire.

Agreement

◯ I agree with the Data Privacy Policy

---

01 In what year was your institution founded?

(Please enter year only (format YYYY - e.g. 1998))

(Any notes?)

02 What scope of services does your facility offer?

- ◯ study coordination only (without patient contact)
- ◯ both study coordination and study execution (including patient contact)

(Any notes?)

03 How many employees does your CTU/CTC have?

(Please enter number only)

(Any notes?)

04 How many full-time equivalents does that equal?

(Please enter number only (use ".", not "," if you use decimals))

(Any notes?)

05 What (estimated) percentage of the trials coordinated last year (Jan. 2020 - Dez. 2020) were investigator-initiated?

0% < - > 100%

(Place a mark on the scale above)

(Any notes?)

Please mark whether your institution covers the following areas with in-house work force...

		Complete coverage	Not covered	Partly covered (please specify below)
1	...monitoring	○	○	○

(Any notes?)

		Complete coverage	Not covered	Partly covered (please specify below)
2	...biostatistics	○	○	○

(Any notes?)

		Complete coverage	Not covered	Partly covered (please specify below)
3	...contract management	○	○	○

(Any notes?)

		Complete coverage	Not covered	Partly covered (please specify below)
4	...intellectual property / technology transfer	○	○	○

(Any notes?)

		Complete coverage	Not covered	Partly covered (please specify below)
5	...quality management	○	○	○

(Any notes?)

		Complete coverage	Not covered	Partly covered (please specify below)
6	...regulatory / legal affairs	○	○	○

(Any notes?)

		Complete coverage	Not covered	Partly covered (please specify below)
7	...budget management	◯	◯	◯

(Any notes?)

		Complete coverage	Not covered	Partly covered (please specify below)
8	...payments management	◯	◯	◯

(Any notes?)

		Complete coverage	Not covered	Partly covered (please specify below)
9	...ethics affairs	◯	◯	◯

(Any notes?)

		Complete coverage	Not covered	Partly covered (please specify below
10	...research consultation	◯	◯	◯

(Any notes)

		Complete coverage	Not covered	Partly covered (please specify below
11	...data management	◯	◯	◯

(Any notes?)

		Complete coverage	Not covered	Partly covered (please specify below
12	...education / training	◯	◯	◯

____
(Any notes?)

		Complete coverage	Not covered	Partly covered (please specify below
13	...feasibility assessment	◯	◯	◯

____
(Any notes?)

		Complete coverage	Not covered	Partly covered (please specify below
14	...protocol development	◯	◯	◯

____
(Any notes?)

		Complete coverage	Not covered	Partly covered (please specify below
15	...project management	◯	◯	◯

____
(Any notes?)

		Complete coverage	Not covered	Partly covered (please specify below
16	...medical writing	◯	◯	◯

____
(Any notes?)

		Complete coverage	Not covered	Partly covered (please specify below)
17	...publication of trial results	◯	◯	◯

____
(Any notes?)

		Complete coverage	Not covered	Partly covered (please specify below
18	...study documents archiving	◯	◯	◯

(Any notes?)

		Complete coverage	Not covered	Partly covered (please specify below
19	...safety management / vigilance	◯	◯	◯

(Any notes?)

		Complete coverage	Not covered	Partly covered (please specify below
20	...IT support / IT development	◯	◯	◯

(Any notes?)

		Complete coverage	Not covered	Partly covered (please specify below
21	...supply of study nurses without patient contact	◯	◯	◯

(Any notes?)

		Complete coverage	Not covered	Partly covered (please specify below
22	...supply of study nurses with patient contact	◯	◯	◯

(Any notes?)

23 If you can think of any further services provided, please mention them here

The following services ARE implemented by your CTU. How important do you consider these services to be for the effective work of your CTU?

	Essential	Quite important	Moderately important	Slightly important	Not important
...monitoring	○	○	○	○	○

(Any notes?) ______________________

	Essential	Quite important	Moderately important	Slightly important	Not important
...biostatistics	○	○	○	○	○

(Any notes?) ______________________

	Essential	Quite important	Moderately important	Slightly important	Not important
...contract management	○	○	○	○	○

(Any notes?) ______________________

	Essential	Quite important	Moderately important	Slightly important	Not important
...intelectual property / technology transfer	○	○	○	○	○

(Any notes?) ______________________

	Essential	Quite important	Moderately important	Slightly important	Not important
...quality management	○	○	○	○	○

(Any notes?) ______________________

	Essential	Quite important	Moderately important	Slightly important	Not important
...regulatory / legal affairs	○	○	○	○	○

(Any notes?)

	Essential	Quite important	Moderately important	Slightly important	Not important
...budget management	○	○	○	○	○

(Any notes?)

	Essential	Quite important	Moderately important	Slightly important	Not important
...payments management	○	○	○	○	○

(Any notes?)

	Essential	Quite important	Moderately important	Slightly important	Not important
...ethics affairs	○	○	○	○	○

(Any notes)

	Essential	Quite important	Moderately important	Slightly important	Not important
...research consultation	○	○	○	○	○

(Any notes?)

	Essential	Quite important	Moderately important	Slightly important	Not important
...data management	○	○	○	○	○

(Any notes?)

	Essential	Quite important	Moderately important	Slightly important	Not important
...education / training	◯	◯	◯	◯	◯

(Any notes?)

	Essential	Quite important	Moderately important	Slightly important	Not important
...feasibility assessment	◯	◯	◯	◯	◯

(Any notes?)

	Essential	Quite important	Moderately important	Slightly important	Not important
...protocol development	◯	◯	◯	◯	◯

(Any notes?)

	Essential	Quite important	Moderately important	Slightly important	Not important
...project management	◯	◯	◯	◯	◯

(Any notes)

	Essential	Quite important	Moderately important	Slightly important	Not important
...medical writing	◯	◯	◯	◯	◯

(Any notes?)

	Essential	Quite important	Moderately important	Slightly important	Not important
...publication of trial results	◯	◯	◯	◯	◯

(Any notes?)

	Essential	Quite important	Moderately important	Slightly important	Not important
...study documents archiving	○	○	○	○	○

(Any notes?)

	Essential	Quite important	Moderately important	Slightly important	Not important
...safety management / vigilance	○	○	○	○	○

(Any notes?)

	Essential	Quite important	Moderately important	Slightly important	Not important
...IT support / IT development	○	○	○	○	○

(Any notes?)

	Essential	Quite important	Moderately important	Slightly important	Not important
...supply of study nurses without patient contact	○	○	○	○	○

(Any notes?)

	Essential	Quite important	Moderately important	Slightly important	Not important
...supply of study nurses with patient contact	○	○	○	○	○

(Any notes?)

The following services are NOT implemented by your CTU. Compared to other possible services, how important do you consider implementing them is to increase the effectiveness of work of your CTU in the future?

	Essential	Quite important	Moderately important	Slightly important	Not important
...monitoring	◯	◯	◯	◯	◯

(Any notes?)

	Essential	Quite important	Moderately important	Slightly important	Not important
...biostatistics	◯	◯	◯	◯	◯

(Any notes?)

	Essential	Quite important	Moderately important	Slightly important	Not important
...contract management	◯	◯	◯	◯	◯

(Any notes?)

	Essential	Quite important	Moderately important	Slightly important	Not important
...intellectual property / technology transfer	◯	◯	◯	◯	◯

(Any notes?)

	Essential	Quite important	Moderately important	Slightly important	Not important
...quality management	◯	◯	◯	◯	◯

(Any notes?)

	Essential	Quite important	Moderately important	Slightly important	Not important
...regulatory / legal affairs	○	○	○	○	○

(Any notes?)

	Essential	Quite important	Moderately important	Slightly important	Not important
...budget management	○	○	○	○	○

(Any notes?)

	Essential	Quite important	Moderately important	Slightly important	Not important
...payments management	○	○	○	○	○

(Any notes?)

	Essential	Quite important	Moderately important	Slightly important	Not important
...ethics affairs	○	○	○	○	○

(Any notes?)

	Essential	Quite important	Moderately important	Slightly important	Not important
...research consultation	○	○	○	○	○

(Any notes?)

	Essential	Quite important	Moderately important	Slightly important	Not important
...data management	○	○	○	○	○

(Any notes?)

	Essential	Quite important	Moderately important	Slightly important	Not important
...education / training	◯	◯	◯	◯	◯

(Any notes?)

	Essential	Quite important	Moderately important	Slightly important	Not important
...feasibility assessment	◯	◯	◯	◯	◯

(Any notes?)

	Essential	Quite important	Moderately important	Slightly important	Not important
...protocol development	◯	◯	◯	◯	◯

(Any notes?)

	Essential	Quite important	Moderately important	Slightly important	Not important
...project management	◯	◯	◯	◯	◯

(Any notes?)

	Essential	Quite important	Moderately important	Slightly important	Not important
...medical writing	◯	◯	◯	◯	◯

(Any notes?)

	Essential	Quite important	Moderately important	Slightly important	Not important
...publication of trial results	◯	◯	◯	◯	◯

(Any notes?)

	Essential	Quite important	Moderately important	Slightly important	Not important
...study documents archiving	◯	◯	◯	◯	◯

(Any notes?)

	Essential	Quite important	Moderately important	Slightly important	Not important
...safety management / vigilance	◯	◯	◯	◯	◯

(Any notes?)

	Essential	Quite important	Moderately important	Slightly important	Not important
...IT support / IT development	◯	◯	◯	◯	◯

(Any notes?)

	Essential	Quite important	Moderately important	Slightly important	Not important
...supply of study nurses without patient contact	◯	◯	◯	◯	◯

(Any notes?)

	Essential	Quite important	Moderately important	Slightly important	Not important
...supply of study nurses with patient contact	◯	◯	◯	◯	◯

(Any notes?)

The following services are PARTLY implemented by your CTU. Compared to other possible services, how important do you consider strengthening them is to increase the effectiveness of work of your CTU in the future?

	Essential	Quite important	Moderately important	Slightly important	Not important
...monitoring	◯	◯	◯	◯	◯

____
(Any notes?)

	Essential	Quite important	Moderately important	Slightly important	Not important
...biostatistics	◯	◯	◯	◯	◯

____
(Any notes?)

	Essential	Quite important	Moderately important	Slightly important	Not important
...contract management	◯	◯	◯	◯	◯

____
(Any notes?)

	Essential	Quite important	Moderately important	Slightly important	Not important
...intellectual property / technology transfer	◯	◯	◯	◯	◯

____
(Any notes?)

	Essential	Quite important	Moderately important	Slightly important	Not important
...quality management	◯	◯	◯	◯	◯

____
(Any notes?)

	Essential	Quite important	Moderately important	Slightly important	Not important
...regulatory / legal affairs	◯	◯	◯	◯	◯

(Any notes?)

	Essential	Quite important	Moderately important	Slightly important	Not important
...budget management	◯	◯	◯	◯	◯

(Any notes?)

	Essenstial	Quite important	Moderately important	Slightly important	Not important
...payments management	◯	◯	◯	◯	◯

(Any notes?)

	Essential	Quite important	Moderately important	Slightly important	Not important
...ethics affairs	◯	◯	◯	◯	◯

(Any notes?)

	Essential	Quite important	Moderately important	Slightly important	Not important
...research consultation	◯	◯	◯	◯	◯

(Any notes?)

	Essential	Quite important	Moderately important	Slightly important	Not important
...data management	◯	◯	◯	◯	◯

(Any notes?)

	Essential	Quite important	Moderately important	Slightly important	Not important
...education / training	○	○	○	○	○

(Any notes?)

	Essential	Quite important	Moderately important	Slightly important	Not important
...feasibility assessment	○	○	○	○	○

(Any notes?)

	Essential	Quite important	Moderately important	Slightly important	Not important
...protocol development	○	○	○	○	○

(Any notes?)

	Essential	Quite important	Moderately important	Slightly important	Not important
...project management	○	○	○	○	○

(Any notes?)

	Essential	Quite important	Moderately important	Slightly important	Not important
...medical writing	○	○	○	○	○

(Any notes?)

	Essential	Quite important	Moderately important	Slightly important	Not important
...publication of trial results	○	○	○	○	○

(Any notes?)

	Essential	Quite important	Moderately important	Slightly important	Not important
...study documents archiving	◯	◯	◯	◯	◯

(Any notes?)

	Essential	Quite important	Moderately important	Slightly important	Not important
...safety management / vigilance	◯	◯	◯	◯	◯

(Any notes?)

	Essential	Quite important	Moderately important	Slightly important	Not important
...IT support / IT development	◯	◯	◯	◯	◯

(Any notes?)

	Essential	Quite important	Moderately important	Slightly important	Not important
...supply of study nurses without patient contact	◯	◯	◯	◯	◯

(Any notes?)

	Essential	Quite important	Moderately important	Slightly important	Not important
...supply of study nurses with patient contact	◯	◯	◯	◯	◯

(Any notes?)

You have reached the end of the questionnaire and now have the opportunity to either review your entries by clicking "previous page" or to finalize the questionnaire later by clicking "save and return later". If you are satisfied with your input, click the "Submit" button below.

If you have any final suggestions, notes or comments, please note them in the text box provided here:

Thank you for your time!

Follow-up Survey:

Dear ICN-Member and CTU Representative,

Last year you participated in a survey of the ICN as a representative of your CTU/CTC on the topic of Clinical Trials Services. We would like to take this opportunity to thank you for your participation!

In the meantime, we have evaluated the survey results and are about to submit a manuscript to a journal. During the evaluation, we noticed that we are missing some information that would help us to better evaluate and contextualize the collected data.

At this point, we would like to ask you for some more information about yourself as a CTU representative and about your institution.

The privacy policy you already agreed to is still valid, i.e. it also covers this extension of the questionnaire. We will publish only aggregated data that does not allow a re-identification of you as an individual or of your organization.

We would very much appreciate your answers to the following questions:

1. What position do you currently hold in your CTU? Please tell us your profession (job title).

2. How many years have you been involved in the field of clinical research/trials yourself?

3. Are you yourself involved in study design, planning or conduct (Yes/No)?

   If Yes: How many studies have you supervised/supported (estimate)?

About your CTU:

Of the total number of studies currently conducted by/with your CTU/CTC, what percentage are

- Drug studies:
- Medical device studies:
- Other studies:

- Monocentric:
- Multicentric:

- National:
- International:

Appendix: Statement on excluded participant

We analyzed 15 responses out of the 16 responses received from ICN-CTU-representatives. We excluded one CTU due to statements which either disqualified the respondent from our intended peer group or/and can be considered contradictory in themselves. We here list only a few statements to exemplify the unsuitability of the responses for being considered for further analysis:

The respondent states that "his/her" CTU consists of only one employee. Physicians/researchers then employ clinical trial managers and other personnel individually for their project. This in itself defies the idea of a Clinical Trial Unit, offering central support.

The questions about service coverage have been answered as: Full in-house coverage of "contract management", "intellectual property/technology transfer", "quality management", "regulatory/legal affairs", "budget management", "payments management", "ethic affairs", "education/training", "archiving", "safety management / vigilance", "IT support / IT development". It seems highly unlikely that one person covers all these areas by him-/herself. And if he/she does, it might be possible for small individual trials only but not for a CTU, which defines itself by offering central support to clinicians of the parent institution and thus hosts multiple trials. Moreover, the responses in the free text field contradict the stated full coverage by declaring, "this [service] is not done by the CTU but is done by the organization".
The respondent thus failed to adhere to our instruction "The questions aim at surveying the services of your facility, not the services of the parent institution (e.g. University Hospital)".

Additionally, when asked about the scope of services offered by the CTU, the respondent declared that the CTU performs both study coordination as well as study execution (including patient contact). Again, in the light of the previous answers this suggests that the respondent has answered not as a CTU but as a hospital representative, as this work cannot be done by one person only. And if it can, it defies our understanding of a CTU.

It seems that the respondent had much misunderstanding on the survey questions and gave many contradicting or unreasonable answers, which could not possibly be rectified by a few simple queries. We therefore concluded that his/her answers would mislead the readers and should be excluded.
